# Supplementary material for: Evaluation of thrombophilia testing in the inpatient setting: A single institution retrospective review
Source: PLoS One. 2021 Sep 20;16(9):e0257687. doi: 10.1371/journal.pone.0257687 (PMC8452015; doi:10.1371/journal.pone.0257687)
Supplement: Attachment — Submitted filename: Response to Reviewers.docx [file pone.0257687.s002.docx]

Corresponding Author: Chun Siu

**Response to reviewers**

**Comments by Reviewer #1**

1) You write that "duration of anticoagulation may not always be affected by results of thrombophilia testing". You should rather state that duration is barely affected by thrombophilia testing with the exceptions of severe thrombophilia and antiphospholipid syndrome

**Response**: We revised the statement so that there are exceptions

2) Use only one consistent abbreviation for anticoagulant sensitive aPTT (you use both LUA and LA). I would suggest to just use "aPTT" because "LA" stands for "Lupus anticoagulant" that can be detected with both aPTT and dRVVT.

**Response:** Revised to ensure consistent abbreviation

3) Antiphospholipid antibodies are often only transiently positive. You should not use the term "false-postive" in this context.

**Response:** Revised terminology

4) Use antithrombin activity instead of antithrombin III

**Response:** Revised terminology

5) "dilute Russell's viper venom": the test is in fact named "dilute Russell's Viper Venom TIME (dRVVT)

**Response:** Revised terminology

6) Given your data, you should at least discuss whether testing for hereditary thrombophilia (not APS!) in an inpatient setting is necessary at all. The most efficient way to reduce costs would be to just avoid inpatient testing for hereditary thrombophilia

**Response:**  Thank you for the feedback. We agree that hereditary thrombophilia should be discussed in more detail so we added that to the manuscript.

7) Are your institution guidelines for thrombophilia testing valid for all hospital services or do some specific services (eg neurology) use their own guidance?

**Response:**  We added into manuscript that our institution guidelines are valid for all hospital services.

**Comments by Reviewer #2**

1) Abstract: The background can be sharpened and maybe even stated in one sentence. Please state the aim of the study. Please report details (Setting, inclusion and exclusion criteria more explicitly). Definition of "Thrombophilia testing" How where the data collected? What was the criterion for "inappropriate" testing? Testing "did not change management". How was this measured?

**Response:**  Revised abstract to improve the presentation of information. We also made revisions based on the comments to further sharpen our abstract.

2) Introduction: (1) What is the problem? You are correct that inpatient thrombophilia testing is often inadequate but is this sufficient to do the study?

**Response:**  Revised abstract to improve how the study problem is presented. The purpose of this study is to highlight the overuse of inpatient thrombophilia testing. We hope that sharing these results will help PLOS One readers make informed decisions on when to do testing.

3) Introduction:(2) What are aims and objectives of the study (this must match the problem stated before)? Please be precise and specific.

**Response:**  We made revisions to emphasize the objectives of this study.

4) Methods: The methods are described very shortly and not very clearly arranged. I suggest including sub-headings (Design, setting and population/ definition of diagnoses / definition of inapropriate testing / data collection / determination of healthcare costs/ statistical analysis). Please follow the STROBE reporting guidelines.
   Regarding data collection: how was the chart review done? How where the patients screened?
   Database query for patient screening: is it valid and complete?

**Response:**  Appreciate the suggestion for sub-headings. Added for better outline of the manuscript. We discussed the data collection process in further detail.

5) Results: I suggest sub-heading to improve clarity (patient characteristics...)
   How many patients were admitted with VTE or arterial thrombosis (overall) and what was the proportion of patients with thrombophilia testing. This would be easy to determine and would strengthen the manuscript.

**Response:**  Appreciate the suggestion for sub-headings. Added total number of pts admitted to our hospital during 2019, which was the study time period.

6) The discussion would benefit from structuring. An established structure is: (1) What did we find? (2) discussion of the results in context of previous literature (3) strengths and limitations (4) what does it mean (5) conclusions.

**Response:** For improved clarity, we restructured the discussion section.

7) Conclusions: "In summary, this retrospective study showed that inappropriate thrombophilia testing is common in the inpatient setting." Please modify this first sentence to make it clear that it only refers to your institution.
   "Based on published retrospective studies, this issue is also seen in other institutions as well." This sentence fits into the discussion, not the conclusion. Please re-word.
   "Inpatient thrombophilia testing rarely affects acute management and should be recommended only in selected patients." Was this observed in your study??
   "The ordering practices revealed the overuse of thrombophilia lab panels.." Was this observed in your study??

**Response:** Appreciate these comments as it was helpful for brainstorming and revising this section. I believe this section is much improved after this revision.
